# Released lipids regulate Transient Receptor Potential Channel (TRP)-dependent oral cancer pain

**DOI:** 10.1186/s12990-015-0016-3

**Published:** 2015-05-26

**Authors:** Shivani Ruparel, Michelle Bendele, Ashley Wallace, Dustin Green

**Affiliations:** Department of Endodontics, University of Texas Health Science Center at San Antonio, 7703 Floyd Curl Drive, San Antonio, Texas 78229 USA; Department of Neuroscience, University of Texas Health Science Center at San Antonio, San Antonio, Texas USA; Department of Physiology, University of Texas Health Science Center at San Antonio, Texas, USA

**Keywords:** Lipids, TRPV1, TRPA1, Oral squamous cell carcinoma, Cancer pain

## Abstract

**Background:**

Pain in the head neck area is an early symptom in oral cancer, supporting the hypothesis that cancer cells control the activities of surrounding nociceptors at the site of the tumor. Several reports implicate TRPV1 and TRPA1 in cancer pain, although there is a large gap in knowledge since the mechanisms for tumor-induced activation of these TRP receptors are unknown. Interestingly, TRP-active lipids such as linoleic acid, arachidonic acid, hydroxyoctadecadienoic acid and hydroxyeicosatetraenoic acid are significantly elevated in the saliva of oral cancer patients compared to normal patients, supporting a possible linkage between these lipids and oral cancer pain. We therefore hypothesize that oral squamous cell carcinomas release certain lipids that activate TRPV1 and/or TRPA1 on sensory neurons, contributing to the development of oral cancer pain.

**Methods:**

Lipid extracts were made from conditioned media of three human oral squamous cell carcinoma (OSCC) cell lines as well as one normal human oral keratinocytes cell line. These were then injected intraplantarly into rat hindpaws to measure spontaneous nocifensive behavior, as well as thermal and mechanical allodynia. For interventional experiments, the animals were pretreated with AMG517 (TRPV1 antagonist) or HC030031 (TRPA1 antagonist) prior to extract injection.

**Results:**

These studies demonstrate that lipids released from the three OSCC cell lines, but not the normal cell line, were capable of producing significant spontaneous nocifensive behaviors, as well as thermal and mechanical allodynia. Notably each of the cell lines produced a different magnitude of response for each of three behavioral assays. Importantly, pre-treatment with a TRPVI antagonist blocked lipid-mediated nocifensive and thermal hypersensitivity, but not mechanical hypersensitivity. In addition, pre-treatment with a TRPA1 antagonist only reversed thermal hypersensitivity without affecting lipid-induced nocifensive behavior or mechanical allodynia.

**Conclusions:**

These data reveal a novel mechanism for cancer pain and provide strong direction for future studies evaluating the cellular mechanism regulating the TRP-active lipids by OSCC tumors.

## Background

Pain due to cancer is notoriously difficult to treat, adds substantially to the emotional burden of having cancer, and is more frequently observed with certain cancer types such as with head and neck carcinomas (HNC). Indeed, 70–85 % of HNC patients report pain as their major symptom [1–4]. Patients typically report spontaneous pain as well as movement-related pain (i.e., mechanical allodynia) at the primary site of tumor development such as the tongue, floor of the mouth, larynx, oropharynx, hypopharynx and salivary glands [5, 6]. Many patients subsequently require opiates and tolerance rapidly develops for many HNC pain patients [7].

Despite the occurrence of pain in cancer, there is incomplete understanding of the mechanisms by which oral tumors evoke pain. Because pain is often the first symptom of oral cancer, occurring even when the tumor is still quite small in size, it is likely that oral cancer cells control the activities of surrounding nociceptors at the site of the tumor. A major class of trigeminal nociceptors express transient receptor potential (TRP) ion channels [8–10], including TRP vanilloid 1 (TRPV1), which has been implicated in oral cancer pain [7, 11]. However, the mechanism by which TRPV1 is activated during oral cancer is unknown.

Many polyunsaturated fatty acids (PUFA) are known to activate or sensitize TRPV1 [12–19]. However, no prior studies have evaluated the role of these lipids in mediating cancer pain. Interestingly, clinical studies have demonstrated that several TRP-active PUFAs such as linoleic acid, arachidonic acid, hydroxyoctadecadienoic acid (HODE) and hydroxyeicosatetraenoic acid (HETE) are significantly elevated in the saliva of oral cancer patients compared to normal patients, supporting a possible linkage between these lipids and oral cancer pain [20]. In addition, TRPA1 is co-expressed with TRPV1 [21], is also activated by oxidized lipids [12, 22–24], modulates the activity of TRPV1 [25, 26] and may participate in cancer pain [27]. Accordingly, we tested the novel hypothesis that oral squamous cell carcinomas release certain oxidized lipids that activate TRPV1 and TRPA1 on sensory neurons, contributing to the development of oral cancer pain.

## Methods

### Cells lines

Human oral squamous cell carcinoma (OSCC) cell lines; HSC2, HSC3 and HSC4 were purchased from Health Science Research Resources Bank, Japan. These cell lines were established from tumors obtained from male patients with the primary site of tumor being either the floor of the mouth or the tongue [28]. The OSCC cell lines were cultivated and maintained in Dulbecco’s minimal essential medium (DMEM, Life Technologies, Carlsbad, CA) supplemented with glutamine, penicillin/streptomycin (pen-strep) and 10 % fetal bovine serum. The immortalized human normal oral keratinocyte (iNOK) cell line; OKF6-TERT2, was kindly provided by Dr. Cara Gonzales of UTHSCSA. This cell line was maintained in keratinocyte serum-free media (Life Technologies, Carlsbad, CA) supplemented with 25 ug/ml bovine pituitary extract (BPE), 0.2 ng/ml epidermal growth factor (EGF) and 0.4 uM calcium chloride.

### Animals

All protocols were approved by the Institutional Animal Care and Use Committee of the University of Texas Health Science Center at San Antonio. Male Sprague–Dawley rats (Charles River Laboratories, Inc., Wilmington, MA, USA) were used for all experiments. Animals were housed for at least 7 days prior to the experiments.

### Drugs

The TRPV1 antagonist, AMG517 was purchased from Selleckchem (Houston, Texas, USA) and the TRPA1 antagonist, HC030031 was purchased from Tocris (Ellsville, Missouri, USA). Both drugs were diluted in 10 % DMSO/80 % mineral oil for peripheral injections and for systemic injections, AMG517 was diluted in 10 % DMSO/4 % Tween/PBS and HC030031 was diluted in 100 % DMSO.

### Preparation of lipid extracts

Cells (3 × 10^5^) were seeded per well of a 6-well plate. Twenty-four hours later, media was changed to 2 ml of basal minimal essential medium (MEM) without pH indicator, without FBS and supplemented with glutamine and pen-strep for OSCC cells and basal keratinocyte-serum free media with pen-strep but without BPE, EGF or calcium chloride. Following 24 hours, conditioned media was harvested and cells were trypsinized and counted using the TC20 automated cell counter (Bio-Rad). Conditioned media was subjected to lipid extraction using the Folch’s Method [29]. Briefly, 10 ml of chloroform: methanol (2:1) was added to 2 ml of conditioned media and vortexed for 10 sec and incubated on ice for 30 min. The solution was then vortexed again for 10 sec, washed with saline and centrifuged at 8000 RPM for 10 min. The lower chloroform layer was collected and dried under nitrogen gas and stored at −80 °C until further use. Light exposure was minimized during lipid extraction. Immediately prior to experiments, the dried extracts were re-suspended in phosphate buffered saline.

### Behavioral testing

All observers were blinded to treatment allocation. Three behavioral assays were used in the study to measure spontaneous nocifensive as well as evoked nociception in response to noxious stimuli. Spontaneous nocifensive behavior was carried out as described [30], whereas thermal allodynia was measured using the radiant heat test [31] and mechanical allodynia was measured using the dynamic aesthesiometer as described previously [32]. The effect of OSCC-released lipids on nociception was studied by injecting 50 ul of re-suspended lipid extract into the plantar surface of the rat hindpaw. Nocifensive behavior (defined as spontaneous flinching, flexion movements or licking of the treated hindpaw) was recorded every 2 min up to a total of 10 min. Thermal and mechanical thresholds were measured from 10 to 150 min after injection.

To determine the role of TRPV1 and TRPA1 in mediating extract-induced nociceptive behaviors, animals were pretreated with vehicle, AMG517 or HC030031 45 min prior to extract injection. A systemic dose of 100 mg/kg of either drug, subcutaneously underneath the neck, was given for spontaneous nocifensive assays, whereas 100 ug of either drug was injected intraplantarly in the hindpaw for thermal and mechanical threshold measurements. The doses for each drug were selected based on previously published literature [33–36]. Nocifensive behavior was measured for 10 min whereas thermal and mechanical thresholds were measured at the peak time of allodynia; which was 10 min post injection of the lipid extract. Paw withdrawal thresholds for ipsilateral as well as contralateral hindpaws were measured.

### Statistics

Data are presented as mean ± SEM. Statistical analyses were performed using one-way or two-way ANOVA with Sidak’s post-hoc test. A statistically significant difference was defined as *P* < 0.05. Error bars are standard error of the mean (S.E.M).

## Results

### OSCC-released lipids induce nocifensive behavior in rats in a TRPV1-dependent manner

**Fig. 1:**
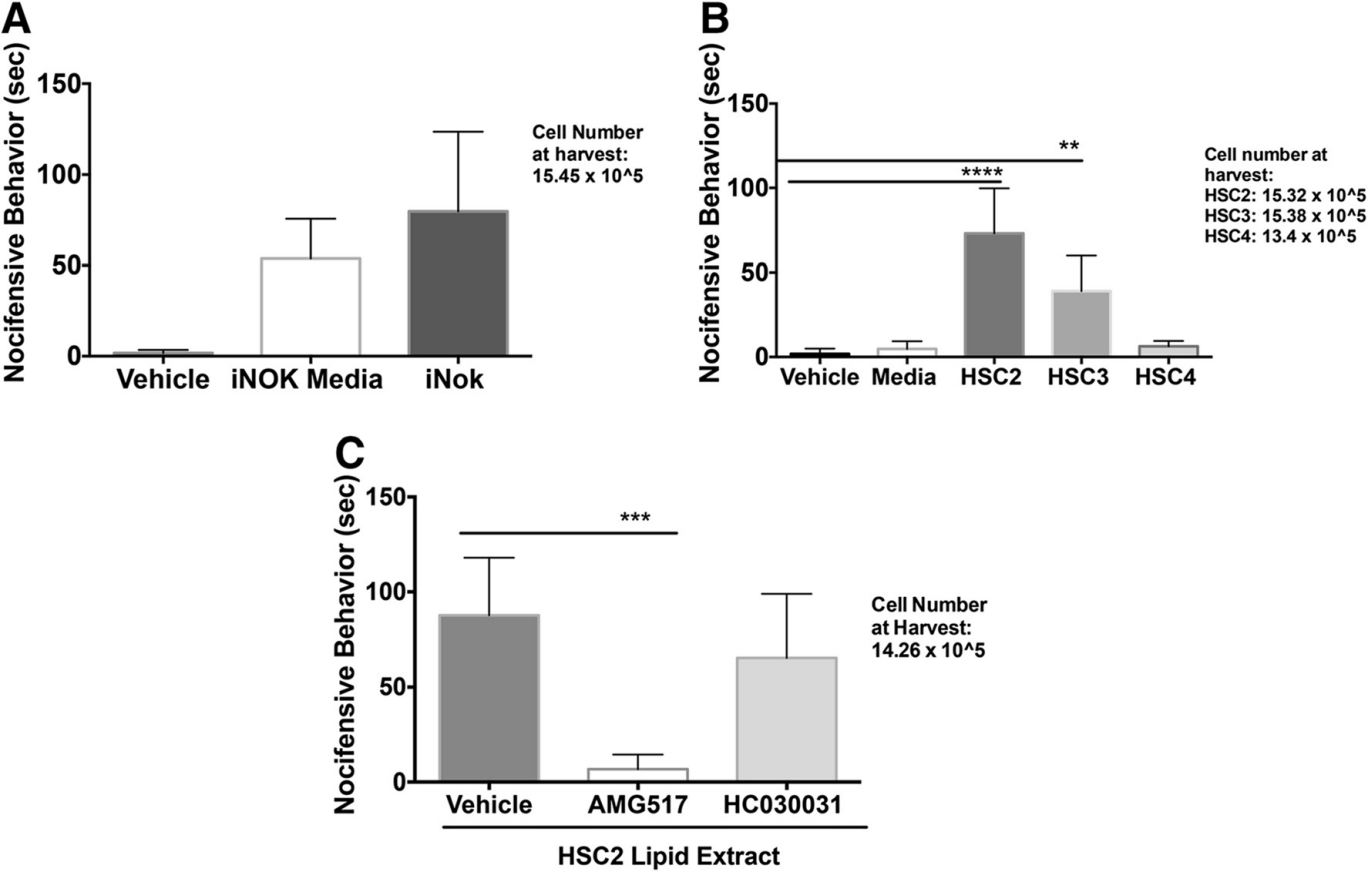
Effect of OSCC-released lipids on nocifensive behavior of rats. Lipid extracts using the Folch’s extraction method were made from conditioned media of (**a**) immortalized normal oral keratinocyte (iNOK) cells and (**b**)*.* HSC2, HSC3 *and* HSC4 cells. 50ul of re-suspended extracts were injected into the plantar surface of rat hindpaw and nocifensive behavior was measured upto 10 min post injection. N = 6/group. Vehicle group was injected with 50ul of PBS. Media group was injected with lipids extracted from media without exposure to cells. Data are represented as mean for each group and error bars indicate SEM. Data were analyzed using two-way ANOVA with Sidak’s post-hoc test with p <0.05. Average cell numbers per well at the time of harvest of conditioned media are indicated. **c** AMG517 (3 mg/kg) or HC030031 (30 mg/kg) was injected subcutaneously underneath the neck and 1 hour later, lipid extract from HSC2 cells was injected in the rat hindpaw and nocifensive behavior measured for 10 min. N =6/group. Data are represented as mean for each group and error bars indicate SEM. Data were analyzed using two-way ANOVA with Sidak’s post-hoc test with p <0.05. Asterisks indicate *p < 0.05; **p < 0.01; ***p < 0.001 and ****p < 0.0001. Average cell numbers per well at the time of harvest of conditioned media are indicated

Because oral cancer patients report spontaneous pain [5, 6], we evaluated the role of OSCC-released lipids in triggering spontaneous nocifensive behavior in rats. Intraplantar injection of lipid extracts from OSCC cell lines HSC2, HSC3 and HSC4 evoked spontaneous nocifensive behaviors in a cell line-dependent manner (Fig. 1b). HSC2 produced the greatest response, followed by HSC3, whereas HSC4 failed to evoke a nocifensive response. The negative controls consisted of phosphate buffered saline (PBS; used to solubilize the lipid extracts) and lipid extracts from cell culture media without exposure to cells; neither of these controls evoked nocifensive behavior. To determine whether the effect of these lipids was specific to cancer cells and not simply a product of oral keratinocytes, we evaluated whether iNOK cells release lipids capable of producing nocifensive behavior. As seen in Fig. 1a, lipids extracts made from iNOK cells did not evoke a nocifensive response more than the extracts from the iNOK growth media alone. The iNOK growth media itself showed a nocifensive response that could be attributed to the basal media constituents required to maintain the cells. Collectively, these data indicate that the nocifensive effects of lipids are specific to OSCC cells.

To determine whether OSCC-released lipids induce nocifensive behavior via activation of TRPV1 and/or TRPA1, rats were pretreated with a systemic dose of specific TRPV1 (AMG517, 3 mg/kg) or TRPA1 antagonist (HC030031, 30 mg/kg) prior to injection of HSC2 lipid extract. As seen in Fig. 1c, AMG517 pretreatment virtually abolished lipid-induced nocifensive behavior, whereas HC030031 pretreatment had no effect.

### OSCC-released lipids induce thermal and mechanical allodynia in rats

**Fig. 2:**
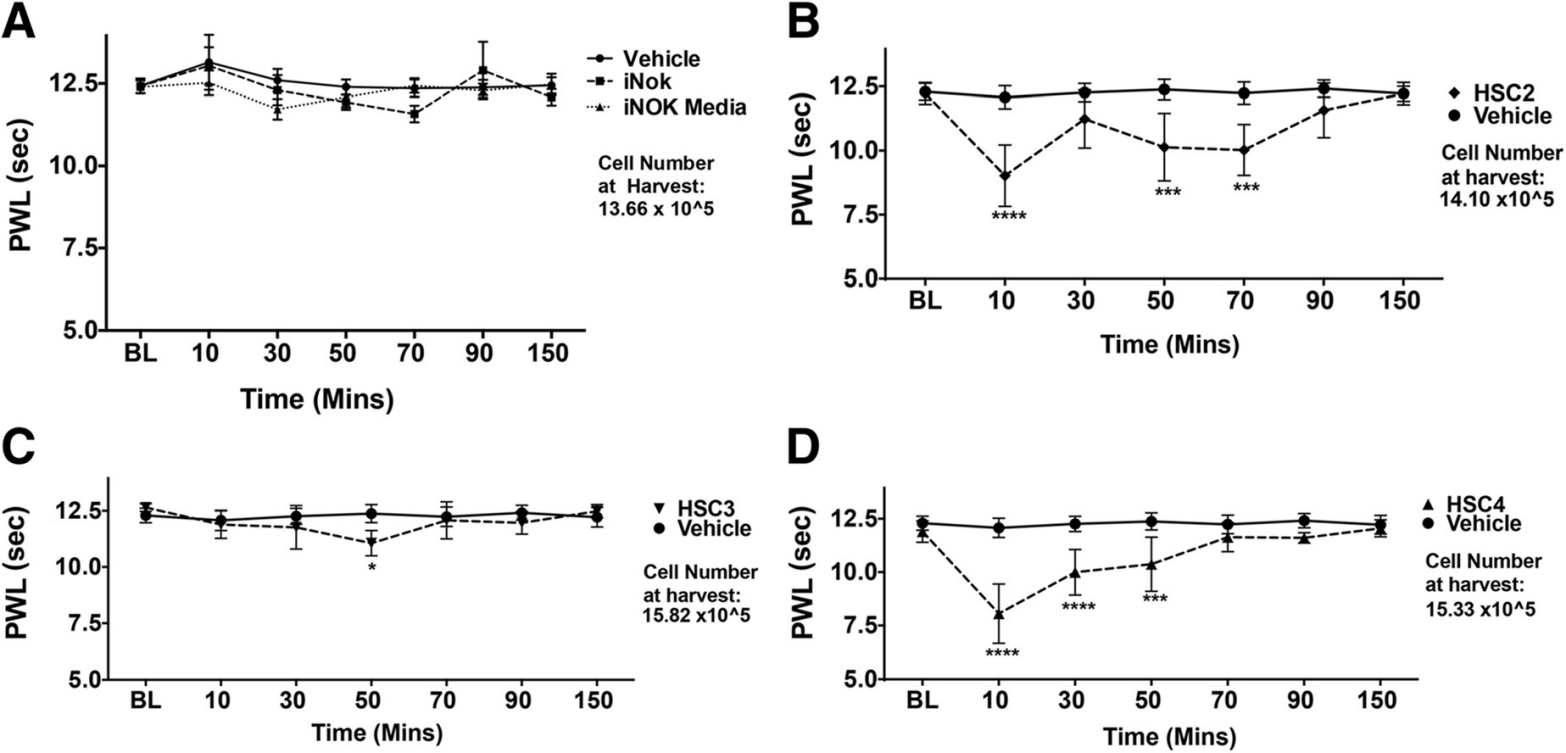
Effect of OSCC-released lipids on thermal thresholds of rats. Lipid extracts using the Folch’s extraction method were made from conditioned media of (**a**)*.* iNOK, (**b**)*.* HSC2, (**c**)*.* HSC3 and (**d**)*.* HSC4 cells. 50ul of re-suspended extracts were injected into the plantar surface of rat hindpaw and thermal thresholds using the radiant heat test were measured up to 150 min post injection. N = 6/group. Vehicle group was injected with 50ul of PBS. Data are represented as mean for each group and error bars indicate SEM. Data were analyzed using two-way ANOVA with Sidak’s post-hoc test with p <0.05. Asterisks indicate *p < 0.05; **p < 0.01; ***p < 0.001 and ****p < 0.0001. Average cell numbers per well at the time of harvest of conditioned media are indicated

**Fig. 3:**
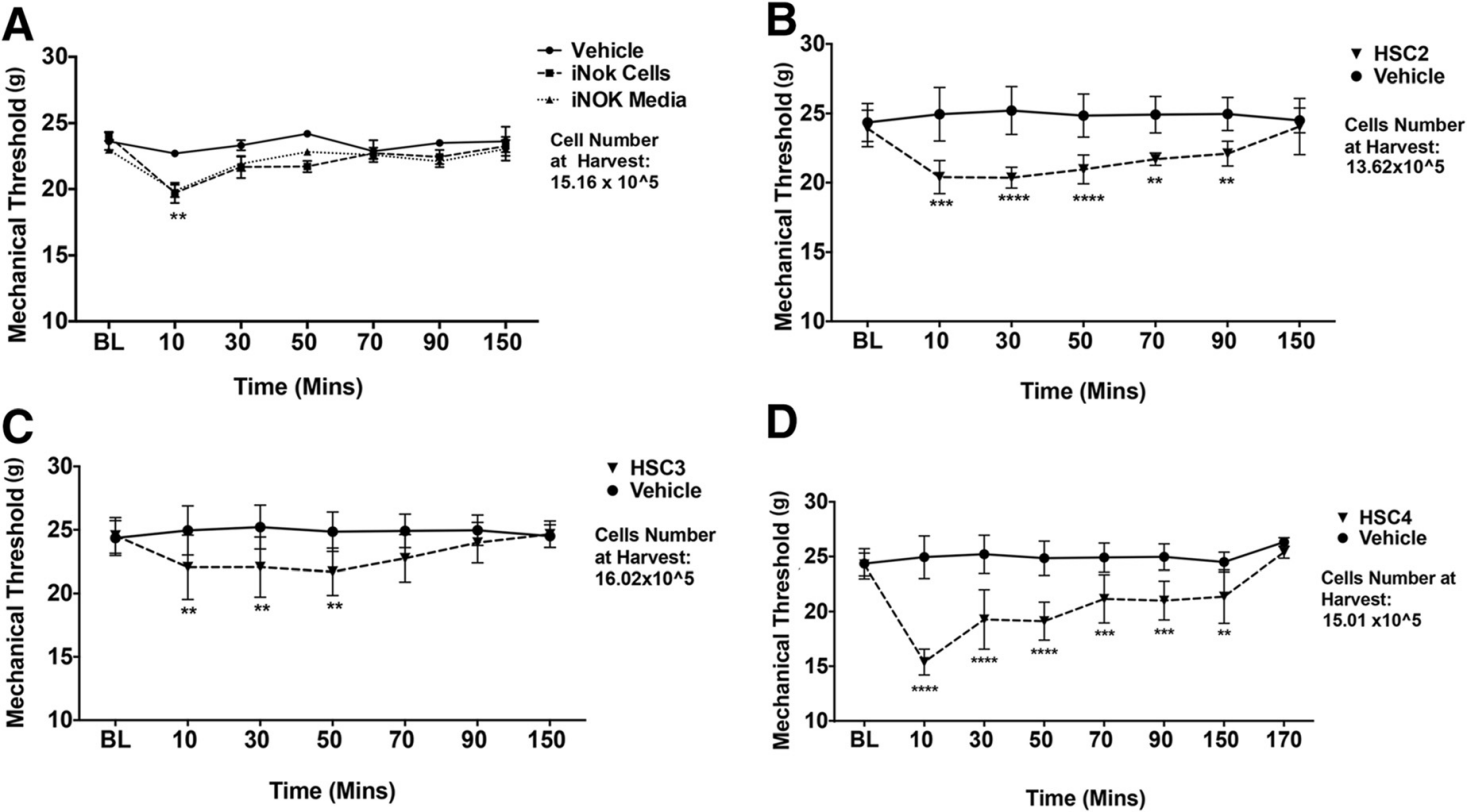
Effect of OSCC-released lipids on mechanical thresholds of rats. Lipid extracts using the Folch’s extraction method were made from conditioned media of (**a**)*.* iNOK, (**b**)*.* HSC2, (**c**)*.* HSC3 and (**d**)*.* HSC4 cells. 50uls of re-suspended extracts were injected into the plantar surface of rat hindpaw and mechanical thresholds using the dynamic aesthesiometer were measured up to 170 min post injection. N =6/group. Vehicle group was injected with 50ul of PBS. Data are represented as mean for each group and error bars indicate SEM. Data were analyzed using two-way ANOVA with Sidak’s post-hoc test with p <0.05. Asterisks indicate *p < 0.05; **p < 0.01; ***p < 0.001 and ****p < 0.0001. Average cell numbers per well at the time of harvest of conditioned media are indicated

We next evaluated the effect of OSCC-derived lipids on thermal and mechanical thresholds. Figure 2b, c and d demonstrated that injection of lipid extracts from HSC2 and HSC4 evoked significant thermal allodynia that lasted up to 50–90 min depending on the cell line. Lipid extracts from HSC3 did not produce a significant reduction in thermal escape thresholds. However, lipid extracts from all three-cell lines evoked significant mechanical allodynia that lasted up to 50–150 min in a cell line-dependent fashion (Fig. 3b, c and d). In contrast, lipids extracted from the iNOK cell line did not evoke significant thermal or mechanical allodynia compared to extracts made from growth media alone (Figs. 2a and 3a).

### OSCC-released lipids mediate thermal but not mechanical allodynia via TRPV1 and TRPA1 channels

**Fig. 4:**
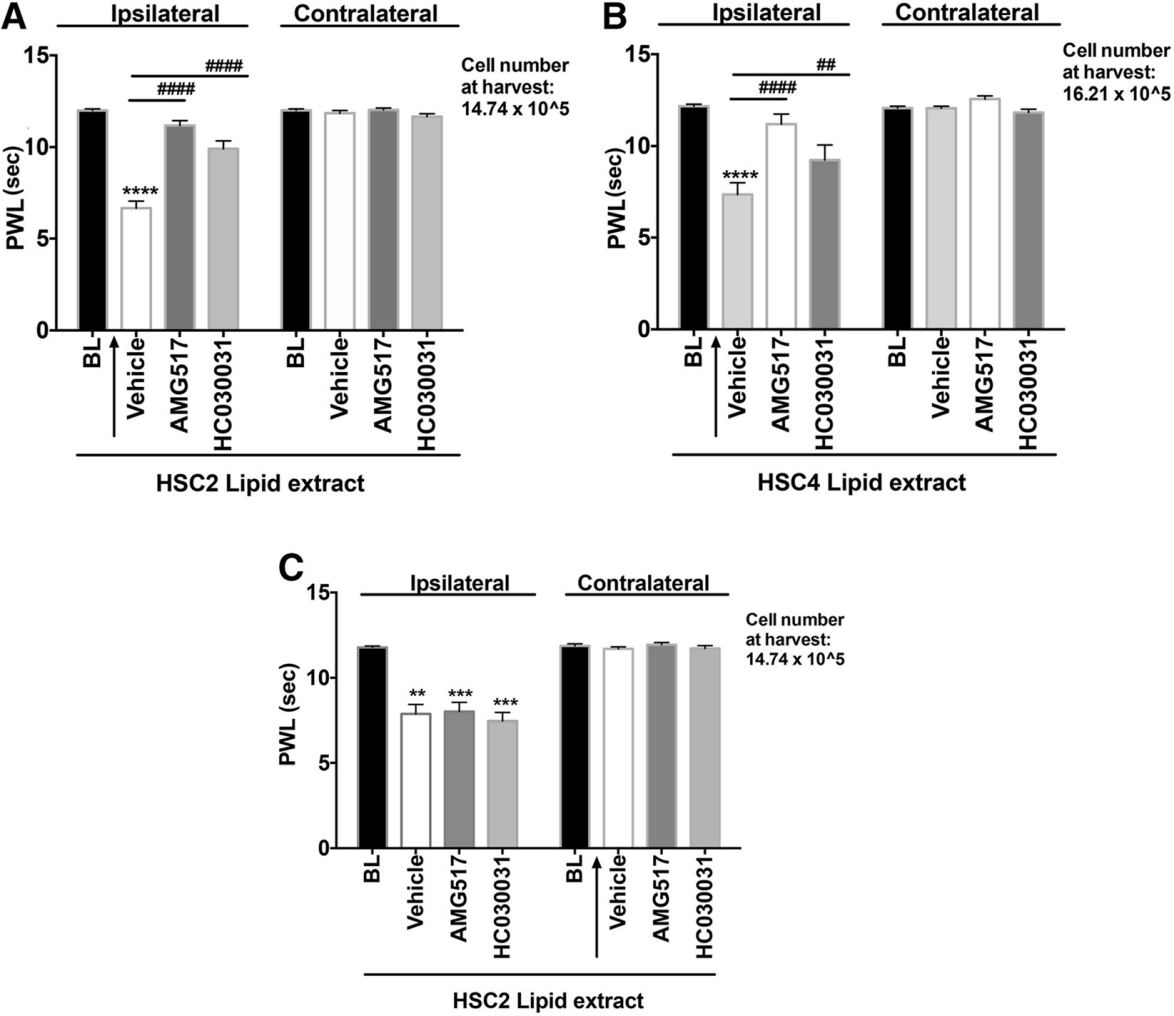
Effect of TRP antagonists on Lipid-induced Thermal Allodynia. TRPV1 antagonist, AMG517 (100 ug) or TRPA1 antagonist HC030031 (100 ug) was injected in the ipsilateral rat hindpaw ipl 45 min prior to lipid extract injection. Thermal threshold were measured 10 min post extract injection of the ipsilateral and contralateral paws. **a** 50ul of re-suspended lipid extract made from HSC2 cells was injected. **b** 50 ul of re-suspended lipid extract made from HSC4 cells was injected. **c** AMG517(100ug) or HC030031(100ug) was injected in the contralateral rat hindpaw ipl and 45 min later, HSC2 lipid extract was injected in the ipsilateral paw. Thermal thresholds were measured 10 min post extract injection of ipsilateral and contralateral paws. N = 6–8 /group. Vehicle group was injected with 50ul of 10%DMSO/90 % Mineral Oil. BL = Baseline thresholds. Data are represented as mean for each group and error bars indicate SEM. Data were analyzed using two-way ANOVA with Sidak’s post-hoc test with p <0.05. Asterisks indicate *p < 0.05; **p < 0.01; ***p < 0.001 and ****p < 0.0001. Average cell numbers per well at the time of harvest of conditioned media are indicated. Arrows indicate the side where vehicle or drug was injected

**Fig. 5:**
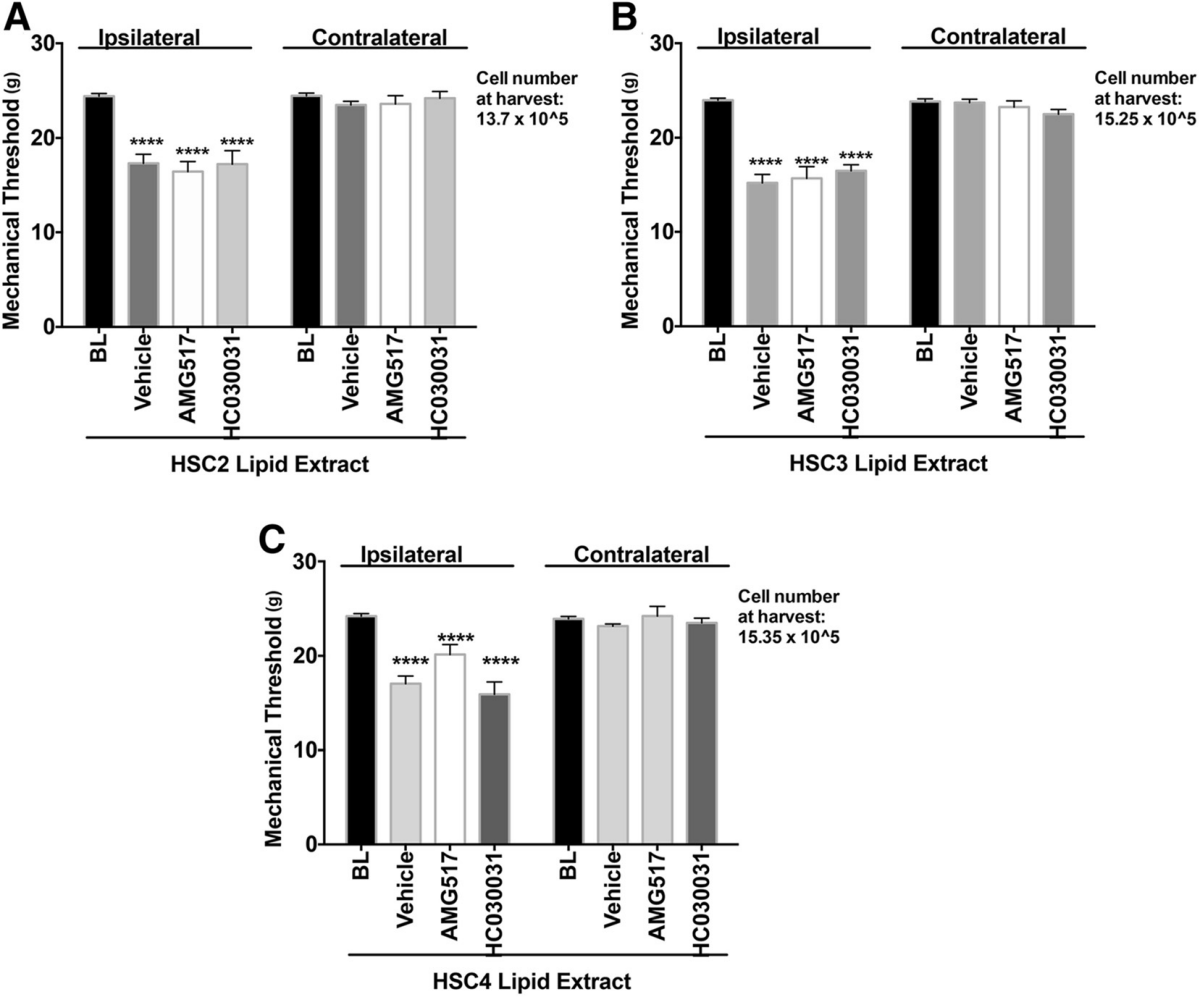
Effect of TRP antagonists on Lipid-induced Mechanical Allodynia. TRPV1 antagonist, AMG517 (100 ug) or TRPA1 antagonist HC030031 (100 ug) was injected in the rat hindpaw ipl 45 min prior to lipid extract injection. Thermal threshold were measured 10 min post extract injection of the ipsilateral and contralateral paws. **a** 50ul of re-suspended lipid extract made from HSC2 cells was injected. **b** 50ul of re-suspended lipid extract made from HSC3 cells was injected. **c** 50ul of re-suspended lipid extract made from HSC4 cells was injected. N = 6–8 /group. Vehicle group was injected with 50ul of 10%DMSO/90 % Mineral Oil. BL = Baseline thresholds. Data are represented as mean for each group and error bars indicate SEM. Data were analyzed using two-way ANOVA with Sidak’s post-hoc test with p <0.05. Asterisks indicate *p < 0.05; **p < 0.01; ***p < 0.001 and ****p < 0.0001. Average cell numbers per well at the time of harvest of conditioned media are indicated

To evaluate whether OSCC-released lipids regulate peripheral activities of TRP channels, rats were pre-treated with intraplantar injection of a vehicle, a TRPV1 antagonist (AMG517) or a TRPA1 antagonist (HC030031) followed by extract injection. Administration of the TRPV1 antagonist reversed lipid-evoked thermal allodynia by 93 % for HSC2 cells and 92 % for HSC4 cells. Similarly pretreatment with the TRPA1 antagonist reduced thermal allodynia by 83 % for HSC2 cells and 76 % for HSC4 cells (Fig. 4). The antagonists did not have an effect on the contralateral paws. To confirm that the effect of antagonist is peripheral and not systemic, we injected AMG517 or HC030031 in contralateral paw and 45 min later tested the effect of extract-induced thermal allodynia in the ipsilateral paw. Figure 4c shows that unlike injection of the TRP antagonist in the ipsilateral paw, injection of antagonists in the contralateral hindpaw had no effect on lipid-induced thermal allodynia. These data indicate that the drug acts locally at the site of injection. The effect of lipids released from HSC3 were not further tested, as the lipid extracts did not produce substantial thermal allodynia. Interestingly, neither the TRPV1 nor the TRPA1 antagonists reversed lipid-induced mechanical allodynia for any of the three cell lines (Fig. 5). Taken together, these data suggests that lipids released by OSCC cells induce thermal allodynia by regulating peripheral TRPV1 and TRPA1 activities but these channels do not mediate lipid-induced peripheral mechanical allodynia.

## Discussion

Two distinct features of pain reports from oral cancer patients are that: 1) the pain is specific to malignant lesions and is not observed in pre-cancerous or non-cancerous lesions [37, 38]; and 2) significant pain occurs even when the tumor is still quite small in size. These aspects of oral cancer pain are consistent with the hypothesis that oral squamous cell carcinoma cells directly modulate activities of peripheral nociceptors. The TRP channels are one class of receptors expressed on the membranes of trigeminal primary afferents terminating in the oral cavity [8–10, 21]. Recent reports have shown that TRPV1 may play a role in oral cancer pain. For example, TRPV1 expression in trigeminal ganglia neurons increases after tumor growth in the oral cavity of rats [7] and ablation of TRPV1^+^ neurons reduced OSCC-induced thermal hyperalgesia [11]. The current study therefore explored the mechanism by which TRPV1 activities are regulated in peripheral sensory neurons during oral cancer pain. Polyunsaturated fatty acids (PUFAs) are a possible class of factors since human saliva from oral cancer patients has been reported to have elevated levels of certain TRP-active PUFAs (e.g., linoleic acid, arachidonic acid, HODE, and HETE), compared to normal control patients [20], and many of these PUFAs activate TRPV1 [12–19] or TRPA1 [12, 22–24]. We therefore evaluated the role of OSCC-secreted lipids in mediating TRP-dependent oral cancer pain using a combined approach of *in vitro* OSCC cell cultures to generate lipid extracts followed by *in vivo* measurement of nociception in rats. This approach focuses on direct effects of tumor cells on peripheral nociceptors without involvement of carcinogenesis, activation of the immune system or alterations in the extracellular matrix.

It has been reported that most HNC patients reporting pain have cancerous lesion in the mouth region [39]. We therefore, used three human OSCC cell lines for our study, namely, HSC2, HSC3 and HSC4, that were derived from tumors obtained from patients with the primary site of tumor being either the floor of the mouth or the tongue [28].

Our data demonstrate that lipids extracted from conditioned media of the OSCC cell lines evoked nocifensive behavior as well as significant thermal and mechanical allodynia (Figs. 1a, 2b, c, and d and 3a, b, and c). Interestingly, the responses observed for each of the three behavioral modalities, were different between the three cell lines. This finding of OSCC heterogeneity may contribute to the observed high variation in pain intensity reported by HNC patients. Importantly, these nociceptive substances appear to be derived from cancerous cells since there were no nociceptive responses observed with lipid extracts from normal oral keratinocyte cells. Moreover, the collection of conditioned media precluded any contribution from other cell types (e.g., immune cells, endothelium, etc.) that might co-exist in a tumor mass grown in vivo. Thus, OSCC release bioactive substances that modulate nociceptor activity. Prior studies have reported that OSCC cells may release certain protein or peptides such as endothelin-1 [40], nerve growth factor (NGF) [41] or trypsin [42], to activate peripheral afferents. However, these and other hydrophilic compounds are excluded by the Folch’s extraction method [29] and thus the present findings demonstrate, for the first time, that lipids released from OSCC cells may contribute to oral cancer pain.

The data also demonstrate a key role for TRPV1 in mediating nociception induced by OSCC-derived lipid extracts. Pre-treatment with a TRPV1 antagonist completely abolished lipid-mediated nocifensive responses and significantly inhibited thermal allodynia. Further, the data indicate that the site of action of the TRPV1 antagonist was peripheral since injection of the drug in the contralateral paw did not affect lipid-induced thermal allodynia. However, TRPV1 is not the only receptor system contributing to lipid-induced nociception. This conclusion is based on two findings. First, a TRPA1 antagonist had significant attenuation of thermal allodynia. Second, neither the TRPV1 nor the TRPA1 antagonist altered mechanical allodynia due to OSCC-derived lipids. Thus, OSCC-released lipids evoke nociception via multiple mechanisms of action. Identifying the specific lipids from OSCC cells can provide further insights into mechanisms by which these lipids induce oral cancer pain as well as, ways to target their release for development of novel analgesics to treat oral cancer pain.
